# Enhanced land use datasets and future scenarios of land change for Slovakia

**DOI:** 10.1016/j.dib.2017.07.066

**Published:** 2017-07-27

**Authors:** Robert Pazúr, Janine Bolliger

**Affiliations:** a WSL Swiss Federal Research Institute, Zürcherstrasse 111, 8903 Birmensdorf, Switzerland; b Institute of Geography, Slovak Academy of Sciences, Štefánikova 49, 814 73 Bratislava, Slovakia

## Abstract

The presented datasets relate to the research article entitled “Land changes in Slovakia: past processes and future directions” [8]. The datasets include the land use and cover (LUC) maps of Slovakia for the years 2006 and 2012 and maps of five future land use scenarios for 2040 developed along the axes of globalisation vs. regionalisation and low vs. high policy intervention (IPCC). Datasets were produced in raster format by combining thematic maps, outputs of models defining particular LUC sector and statistical data taken from European and national predictions of future land change development.The maps have a spatial resolution of 20 m

**Specifications Table** [please fill in right-hand column of the table below]

**Table:** 

Subject area	*Geography, Geoinformation, Landscape Ecology*
More specific subject area	*Land use and land cover classification; future land change scenarios*
Type of data	*raster map, text file, graph*
How data was acquired	*Processing of data sources taken from* EEA [3] and GCI [2]. Data were processed in form of raster maps by using the raster package in R [4], [9].
Data format	*Raster maps (resolution of 20 metres)*
Experimental factors	
Experimental features	
Data source location	*Slovakia*
Data accessibility	
Related research article	*Pazúr and Bolliger*[8]. *Land changes in Slovakia: Past processes and future directions. Applied Geograph, 85, 163–175.*

**Value of the data**

• Thematically and spatially enhanced LUC data provide most detailed and accurate temporal layers of LUC for the years 2006 and 2012 available for Slovakia on national level
• Overlay of LUC dataset for 2006 and 2012 improves on existing knowledge of recent changes in LUC
• Five future land use scenarios for 2040 were developed along the axes of globalisation vs. regionalisation and low vs. high policy intervention.
• Future scenarios provide important baseline information for researchers and practitioners for implementation into management practice and to gain insights into likely magnitudes and locations of land-change in the future.

## 1 Data

**Fig. 1:**
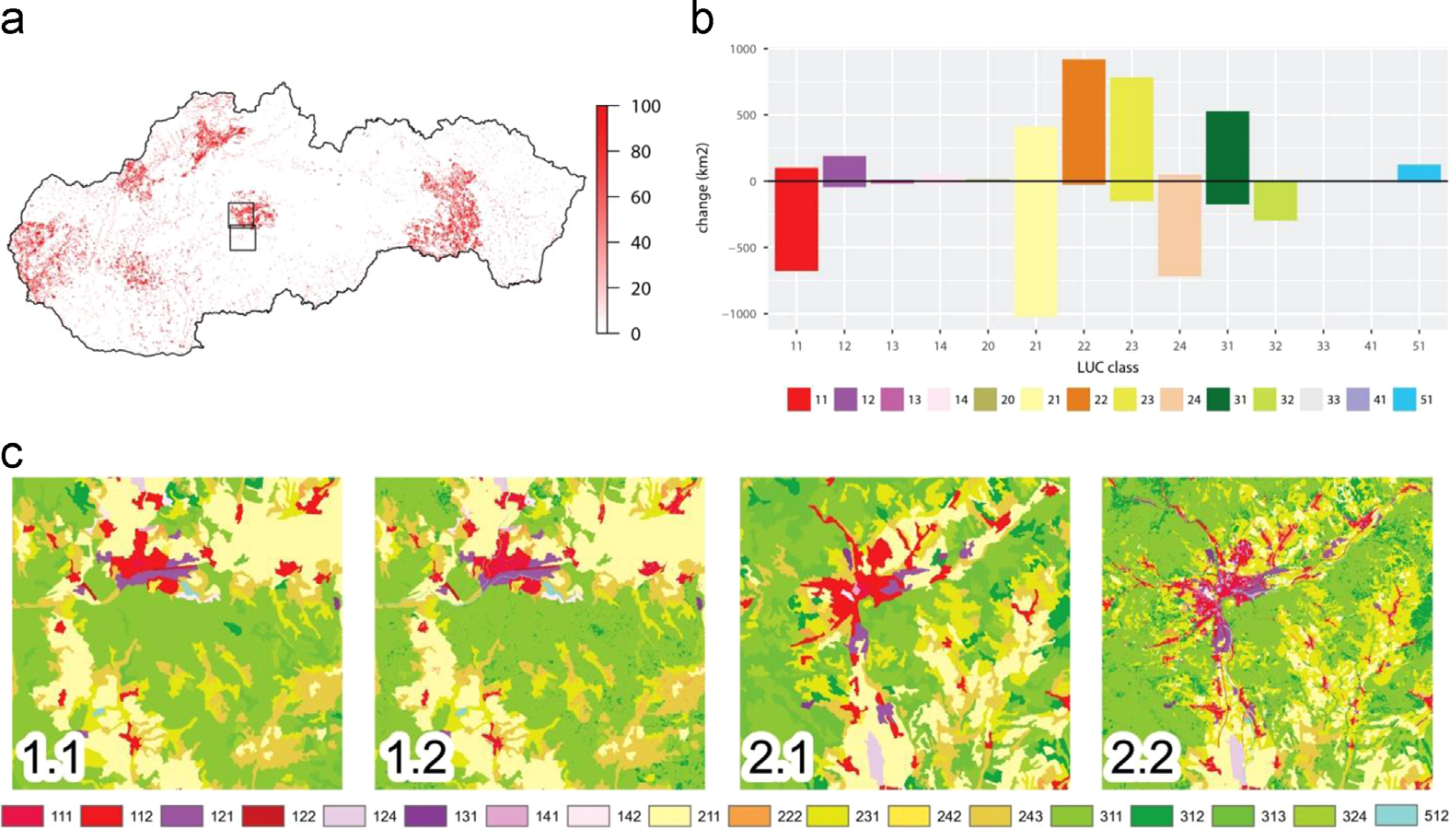
Enhancing representation of land cover classes with additional data: (a) percentage of change within 1 km×1 km grid; (b) comparing the original Corine with the improved land-use classes. The improvement contained additional information from the Urban Atlas, the water body map, ZB GIS and the soil-sealing layer; (c) examples of improvement of the spatial details of land-cover classes (regions marked by rectangles in S1a; 3rd level of classification): (1.) outside and (2.) within the area mapped by Urban Atlas project: (.1) original CORINE land cover map, (.2) refined land cover map. LC codes area similar as in Table 1.

The LUC classification for Slovakia was developed using the existing CORINE dataset for 2006 and 2012. Improvements of the CORINE LUC classification encompassed higher thematic improvement (20 m) relied on supplements regarding settlement structures, agricultural areas, forests and water bodies (see Section 2.1). Supplementary dataset also substantially enhanced the spatial resolution which is in original CORINE LUC dataset limited by minimum mapping units of 25 ha and mapped change areas of 5 ha (Fig. 1).

**Fig. 2:**
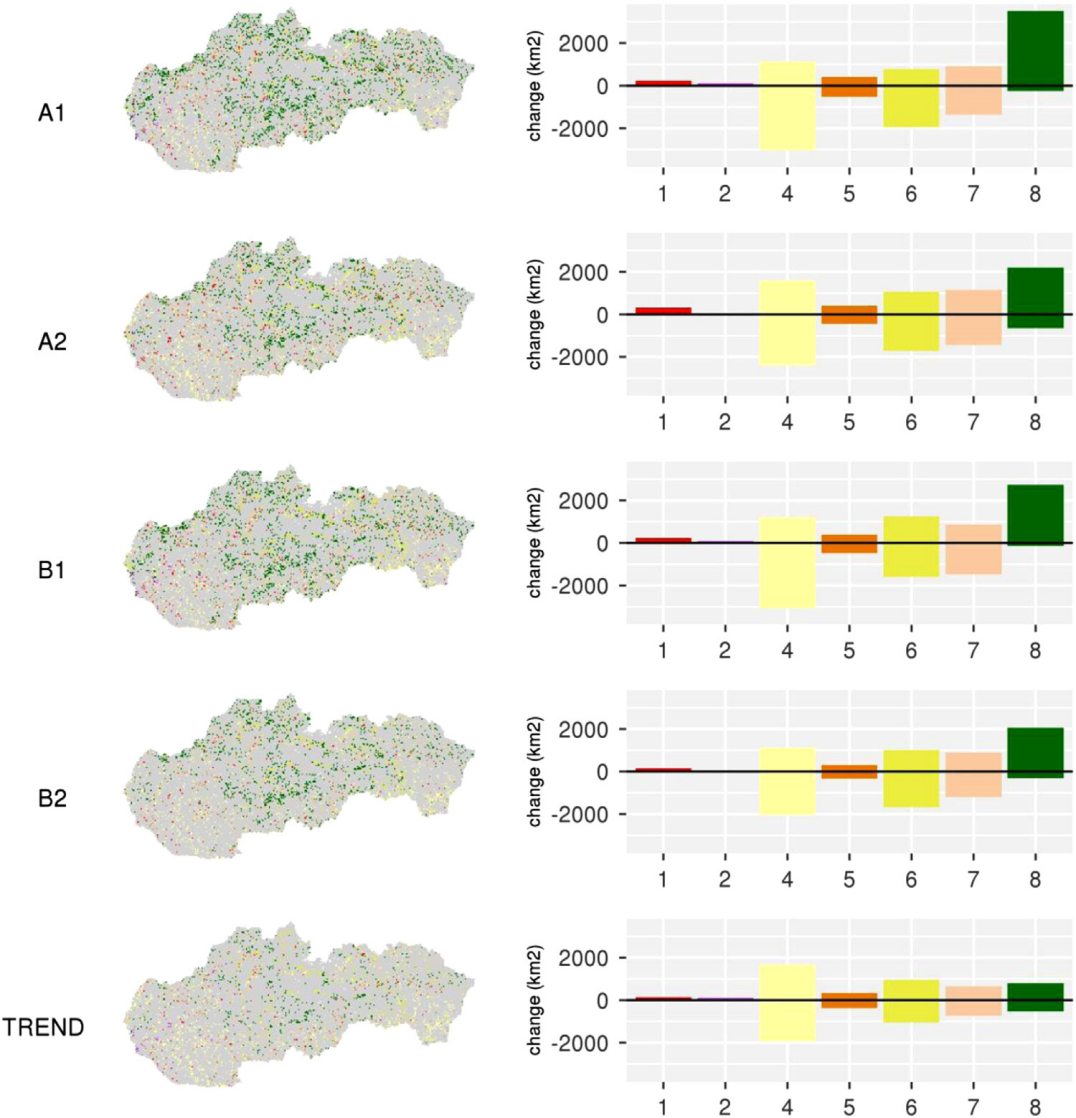
Land change and its allocation among different future land use scenarios. Description of categories is provided in Table 1.

The enhanced LUC datasets were used as an input for developing the spatially explicit future scenarios of land change in Slovakia. Scenarios assumed different development trends as defined by the development pathways from recent years (TREND scenario; [8]), or by storylines compiled from national [6], [7], European [5], [10] or global (IPCC scenarios) assumptions of development along the axes of globalisation vs. regionalisation and low vs. high policy intervention (scenarios A1,A2,B1,B2; Fig. 2, [8]).

## 2 Experimental design, materials and methods

### 2.1 Improving the spatial resolution of land classes

To improve the spatial resolution of urban land cover areas within the CLC dataset, we used the soil sealing layer, which is part of the Pan-European High-Resolution Layers [3] developed within the framework of the Copernicus land monitoring service. Housing, administrative, or industrial buildings localised on non-urban areas with soil sealing greater than 30% were classified either as discontinuous urban fabric or as industrial, commercial and transportation units. Such a distinction depended on the Reference Spatial Database (ZB GIS, the geometric database of the Slovak National infrastructure of spatial data, [2]) which was also used to delineate permanent crops (vineyards, fruit trees and berry plantations CLC class 22) within agricultural fields. Land-use classification of eight major cities in Slovakia classified as Local Administrative Units centres (LAU1, previously called NUTS-4) and their hinterland (so-called Functional Urban Areas) was also improved by using datasets developed by the Urban Atlas project [3]. Combining the CLC with the Urban Atlas (UA) layer, we were able to improve the resolution of the minimum mapping unit (minimum size of 0.25 ha for urbanised areas and 1 ha for non-urbanised areas in UA nomenclature) and identify patches that were not considered within the CORINE nomenclature, such as linear patches (minimum width of linear elements is 10 m in UA nomenclature). To integrate the soil sealing layer and the Urban Atlas into the CLC dataset, we adopted an approach that increased the spatial resolution by updating the CLC dataset in a step-wise manner while maintaining the original CLC nomenclature [1].

For the purpose of refinement, we also used the European waterbody map derived from the Pan-European High-Resolution (HR) datasets. Here, significant water courses and lakes are distinguished, which represent landscape features that are not entirely contained within CLC or the Urban Atlas layers.

The illustration of the 2012 LUC refinement shows that most changes that occurred within those areas that were mapped in the Urban Atlas project (Fig S1a,c) correspond to regional capitals and their hinterland. Updating the CORINE LUC dataset by using the Urban Atlas dataset, ZB GIS and HR waterbody dataset substantially decreases the occurrence of urbanised areas. Most of the urbanised (Table 1).

**Table 1:** CORINE land cover nomenclature. Modified Level 3 classification was used for enhanced LUCC datasets Modified level 2 classification was used for developing the future scenarios. Future scenarios were categorised.

**Level 1**	**Level 2**	**Level 3**	**Category in the scenario model**
1. Artificial surfaces	1.1. Urban fabric	1.1.1. Continuous urban fabric	1
		1.1.2. Discontinuous urban fabric	1
		**1.1.3. Low-density urban fabric**	**1**
		
	1.2. Industrial,commercial and transport units	1.2.1. Industrial or commercial units	2
		1.2.2. Road and rail networks and associated land	2
		1.2.3. Port areas	3
		1.2.4. Airports	3
		**1.2.9. Heterogenous ind., commertial and tran., units**	**2**
	1.3. Mine, dump	1.3.1. Mineral extraction sites and construction sites	3
		1.3.2. Dump sites	3
		1.3.3. Construction sites	3
	1.4. Artificial non-agricultural vegetated areas	1.4.1. Green urban areas	1
		1.4.1. Green urban areas	1
			
2. Agricultural areas	2.1.Arable land	2.1.1. Non-irrigated arable land	4
	2.2. Permanent crops	2.2.1. Vineyards	5
		2.2.2. Fruit trees and berry plantations	5
	2.3. Pastures	2.3.1. Pastures	6
	2.4. Heterogeneous agricultural areas	2.4.1. Annual crops associated with permanent crops	7
		2.4.2 Complex cultivation pattern	7
		2.4.3.Land principally occupied by agriculture, with significant areas of natural vegetation	7
		**2.9.9. Heterogenous non-specified areas**	**7**
			
3. Forests and semi-natural areas	3.1. Forests	3.1.1. Broad-leaved forest	8
		3.1.2. Coniferous forest	8
		3.1.3. Mixed forest	8
		
	3.2. Shrub and/or herbaceous vegetation association	3.2.1. Natural grassland	8
		3.2.2. Moors and heathland	8
		3.2.4. Transitional woodland shrub	8
	3.3. Open spaces with little or no vegetation	3.3.2. Bare rock	9
		3.3.3. Sparsely vegetated areas	9
		3.3.4. Burnt areas	9
		4.1.1. Inland marshes	10
			
4. Wetlands	4.1. Inland wetlands	4.1.2. Peatbogs	10
		5.1.1. Water courses	10
			
5. Water bodies	5.1 Inland waters	5.1.2. Water bodies	10

areas were converted to permanent crops (CLC class 22), which implies the allocation of gardens within cities and their hinterlands in the refined dataset. The opposite change towards urbanised and industrialised areas (CLC class 11 and 12, respectively) was recorded on the small or dispersed settlement patterns which are not able to be mapped by applying the CLC nomenclature (minimum area size of CLC patch is 25 ha). The appearance of small settlement structure negatively influenced the amount of agricultural land, mostly arable land (CLC class 21). Refinement also shrank the heterogeneous agricultural areas (24) which were transformed to more agriculture-specific land uses (arable land, permanent crops or pastures). New forested areas, shrubs and waterbodies appeared in the refined dataset mostly as linear features which did not reach the minimum area size for mapping units in the CLC dataset.

### 2.2 Future scenarios

Future scenarios consist of four general parts that define the spatial policies and restrictions, specific land use conversion settings and land use demand and local suitability assessments [11]. Detailed settings of these parameters as well as determinants used to model the scenario-based outputs are documented in [8]. Spatial extent and location of LUC change under different scenarios, as well as related LUC gains and losses are illustrated in Fig. 2.

## Footnotes

Transparency document Transparency data associated with this article can be found in the online version at 10.1016/j.dib.2017.07.066.
