# Supplementary material for: Enhanced land use datasets and future scenarios of land change for Slovakia
Source: Data Brief. 2017 Jul 27;14:483–8. doi: 10.1016/j.dib.2017.07.066 (PMC5554984; doi:10.1016/j.dib.2017.07.066)
Supplement: Supplementary file 1 — Supplementary material [file mmc1.pdf]

Hao-Ran Wang
Editor-in-Chief
Data in Brief

26th June 2017

CONFLICT OF INTEREST DECLARATION

Title of Paper: **Enhanced land use datasets and future scenarios of land change for Slovakia**

The authors confirm that there are no known conflicts of interest associated with this publication and there has been no significant financial support for this work that could have influenced its outcome.

We also warrant that the manuscript is not under consideration for publication elsewhere.

Robert Pazúr

Janine Bolliger
